# The stabilizing effects of immobilization in D-amino acid oxidase from *Trigonopsis variabilis*

**DOI:** 10.1186/1472-6750-8-72

**Published:** 2008-09-17

**Authors:** Iskandar Dib, Bernd Nidetzky

**Affiliations:** 1 Research Centre Applied Biocatalysis, Petersgasse 14, A-8010 Graz, Austria; 2 Institute of Biotechnology and Biochemical Engineering, Graz University of Technology, Petersgasse 12, A-8010 Graz, Austria

## Abstract

**Background:**

Immobilization of *Trigonopsis variabilis *D-amino acid oxidase (*Tv*DAO) on solid support is the key to a reasonably stable performance of this enzyme in the industrial process for the conversion of cephalosporin C as well as in other biocatalytic applications.

**Results:**

To provide a mechanistic basis for the stabilization of the carrier-bound oxidase we analyzed the stabilizing effects of immobilization in *Tv*DAO exposed to the stress of elevated temperature and operational conditions. Two different strategies of immobilization were used: multi-point covalent binding to epoxy-activated Sepabeads EC-EP; and non-covalent oriented immobilization of the enzyme through affinity of its N-terminal *Strep*-tag to *Strep*-Tactin coated on insoluble particles. At 50°C, the oriented immobilizate was not stabilized as compared to the free enzyme. The structure of *Tv*DAO was stabilized via covalent attachment to Sepabeads EC-EP but concomitantly, binding of the FAD cofactor was weakened. FAD release from the enzyme into solution markedly reduced the positive effect of immobilization on the overall stability of *Tv*DAO. Under conditions of substrate conversion in a bubble-aerated stirred tank reactor, both immobilization techniques as well as the addition of the surfactant Pluronic F-68 stabilized *Tv*DAO by protecting the enzyme from the deleterious effect of gas-liquid interfaces. Immobilization of *Tv*DAO on Sepabeads EC-EP however stabilized the enzyme beyond this effect and led to a biocatalyst that could be re-used in multiple cycles of substrate conversion.

**Conclusion:**

Multi-point covalent attachment of *Tv*DAO on an isoluble porous carrier provides stabilization against the denaturing effects of high temperature and exposure to a gas-liquid interface. Improvement of binding of the FAD cofactor, probably by using methods of protein engineering, would further enhance the stability of the immobilized enzyme.

## Background

D-Amino acid oxidases (DAOs; EC 1.4.3.3) are well characterized flavoenzymes that catalyze oxidative deamination of various α-D-amino acids to the corresponding α-keto acids and ammonia. Molecular oxygen is used as the co-substrate and reduced to H_2_O_2 _in the course of the enzymatic reaction [1-4]. While DAOs show absolute selectivity for conversion of *R*-configured α-amino acids, these enzymes tolerate a wide range of structural variations in the side chain of their substrates. Based on these properties, several technological applications have been suggested for DAOs, including the conversion of amino acid precursors to α-keto acids [5], the resolution of racemic mixtures of (non-natural) amino acids [6,7], and the use in biosensors for quantitative detection of D-amino acids [8-10]. The currently most important application, however, is in the two-step enzymatic conversion of cephalosporin C to 7-amino cephalosporanic acid (7-ACA), a key intermediate for numerous cephem antibiotics [11,12]. The use of DAO in the first step of this biocatalytic reaction is one of the few examples where an oxidizing enzyme was implemented successfully in an industrial process on a multiton-per-year scale. DAO from the yeast *Trigonopsis variabilis *(*Tv*DAO) has good activity towards the sterically demanding substrate [1-4] and exhibits useful stability in the presence of the oxidants (O_2_, H_2_O_2_) occurring in the reaction [11,13]. *Tv*DAO has therefore often been the enzyme of choice in the transformation of cephalosporin C.

A carrier-bound insoluble preparation of *Tv*DAO is used as catalyst for the enzymatic conversion of cephalosporin C [11,12]. The reaction is performed in aerated stirred tank reactors that are operated in a batchwise manner. Immobilization improves the stability of the enzyme under industrial process conditions and enables continuous re-use of *Tv*DAO [14,15]. However, the high costs of current enzyme production and of the employed carrier are major drivers for the improvement of the established immobilization process. Further stabilization of immobilized *Tv*DAO would probably lead to a significant economic benefit.

Various authors have therefore addressed the topic of immobilization of *Tv*DAO on insoluble carriers. Glyoxyl agarose [16,17], aminated polymeric supports [18], or oxirane resins [19] were used to achieve covalent tethering of *Tv*DAO. Surface activation or crosslinking with glutaraldehyde was also applied to accomplish a largely irreversible attachment of *Tv*DAO on various carrier types [16-18,20,21]. The main focus of the majority of these works was of course on the optimization of the immobilization protocol with the goal of achieving maximum stabilization of *Tv*DAO. Multi-point covalent attachment realized through the use of a highly activated carrier or by crosslinking with glutaraldehyde was shown to result in a more pronounced stabilization than immobilization on poorly activated carriers [16] or non-covalent adsorptive bonding [18].

Some authors have obtained remarkable stabilizing effects. López-Gallego et al. [18] for instance report on a 600-fold improvement of stability of *Tv*DAO at high temperature by using a two-step protocol of immobilization where the enzyme was adsorbed on a support coated with polyethylenimine and subsequently treated with glutaraldehyde. In terms of operational stability, an immobilized preparation on glutaraldehyde-activated Duolite could be used in 20 recycles of cephalosporin C conversion without loss of activity [21]. Co-immobilization of *Tv*DAO and catalase resulted in a biocatalyst exhibiting full activity over 40 cycles of α-keto acid production [20]. However, despite these significant successes the quest for new immobilization procedures that provide further improved performance of the carrier-bound *Tv*DAO catalyst with respect to both activity and stability is actively continued in both academia and industry. Unfortunately, only few authors have so far treated the underlying phenomena of stabilization of the carrier-bound oxidase at elevated temperatures [16,18] or under process conditions [17,20]. A comprehensive analysis of the beneficial effects of immobilization on the stability of *Tv*DAO is therefore still missing. Lacking mechanistic understanding of the inactivation of the immobilized enzyme, rational design of an insoluble carrier-bound biocatalyst with improved process performance remains elusive.

We have recently proposed a kinetic mechanism of thermally induced denaturation of *Tv*DAO in solution [22]. Inactivation of the enzyme at 50°C was suggested to proceed via two main pathways: partial loss of protein structure leading to a denatured holoenzyme; and reversible release of FAD cofactor generating inactive apoenzyme. Based on this model we have identified in this paper the underlying molecular processes of thermal inactivation in two different immobilized preparations of *Tv*DAO. Using a miniaturized reactor system, the operational stability of immobilized *Tv*DAO was analyzed and factors that confer additional stability to the enzyme were recognized. The results reported herein provide an improved knowldege basis for the development of stabilized carrier-bound preparations of this industrially important enzyme. Note that method improvement and optimization of the immobilization of *Tv*DAO was not a goal of this work.

## Methods

### Materials and enzymes

Recombinant *Tv*DAO bearing *Strep*-Tag II at its N-terminus was produced in *Escherichia coli *BL21 (DE3) as described previously [23,24]. Purification of the enzyme to apparent electrophoretic homogeneity was done in a single step of affinity chromatography using a *Strep*-Tactin Superflow Cartridge (IBA GmbH, Göttingen, Germany; [23]). The enzyme was stored at -20°C in stock solution containing about 4 mg protein/mL in 10 mM Tris-HCl buffer, pH 7.5.

Standard grade Sepabeads EC-EP with a particle size distribution of 150 – 300 μm (mean diameter: 200 – 240 μm) and an average pore size of 30 – 40 nm were kindly provided by Resindion S.R.L. (Binasco, Italy). The declared dry mass and the specific volume of the beads were 35 – 45% and 2.8 – 3.3 mL/g dry carrier, respectively. The batch number of the beads was SC 1P 114/347. The beads were stored in a well-sealed container at 4°C and used within the shelf-life given by the manufacturer. All other reagents were of analytical grade. They were obtained from Sigma or Fluka and have been described in previous publications [22-24].

### Enzyme immobilization

The enzyme was immobilized on Sepabeads EC-EP basically by following a three-step protocol described elsewhere [25]. All steps were carried out at 18°C. Reaction tubes were gently agitated on an orbital shaker (60 rpm) in horizontal orientation. Hydrophobic adsorption of the enzyme on the carrier was done by using 1 g of carrier and 10 mL of enzyme solution (or multiples thereof) in 500 mM sodium phosphate buffer, pH 7.5. Special care was taken to ensure complete exchange of the original buffer of the enzyme stock solution to phosphate buffer through repeated cycles of concentration and dilution using Vivaspin 6 ultrafiltration tubes (Vivascience, Hannover, Germany). Note that Tris contains amino groups that may react with the epoxy support. Unless otherwise noted the concentration of *Tv*DAO was 0.5 mg/mL. Protein concentration and enzyme activity in the supernatant were followed over a total incubation time of four hours.

For the second step, i.e. the formation of covalent bonds between enzyme and support, buffer was exchanged to 100 mM sodium phosphate, pH 8.5. The reaction was allowed to proceed for 24 h before blocking the free epoxy groups by adding glycine to a final concentration of 1 M. Incubation was then continued for another 14 h. Buffer was removed, beads were washed excessively with 50 mM sodium phosphate buffer, pH 7.5, and stored in this buffer at 4°C until further use.

Directed non-covalent attachment of the enzyme on *Strep*-Tactin MacroPrep particles (IBA GmbH) with an average diameter of 50 μm was achieved by mixing a solution of the enzyme (0.4 mg/mL) with a 50% suspension of the carrier in a 5:1 ratio. Binding was allowed to proceed for 60 min at 20°C with gentle mixing. The absence of unbound protein in the supernatant was confirmed with the Bradford assay and by recording absorption spectra in the range 200–800 nm. Beads were washed twice with Tris-HCl buffer (10 mM, pH 7.5) and stored at 4°C in this buffer.

Any exchange of buffer required for further experiments with the immobilized enzyme preparations was achieved by centrifuging the beads and removing the buffer. Then, the particles were washed twice and finally resuspended in fresh buffer.

### Assays

Activity of soluble *Tv*DAO was routinely determined using a photometric assay in which 2,6-dichloroindophenol (DCIP) replaced dioxygen as the electron acceptor [26]. Measurements were done at 30°C in 100 mM potassium phosphate buffer, pH 8.0, using D-methionine (20 mM) as the substrate.

For immobilized preparations of *Tv*DAO the activity was assayed by measuring the depletion of dissolved oxygen during the enzymatic conversion of D-methionine. A fluorescence-based fiber-optic sensor (PreSens GmbH, Regensburg, Germany) was used to determine O_2 _consumption rates. Oxygen-saturated solutions (100 mM potassium phosphate buffer, pH 8.0) with an O_2 _concentration of around 1,200 μM were employed. A suspension of the immobilized enzyme was stirred at 750 rpm and 30°C in a glass vial that was sealed with a septum (total volume 4.7 mL). The substrate D-methionine was then added through the septum using a syringe to yield a final concentration of 6 mM. The specific activity of immobilized *Tv*DAO (*r*_O2_/[E_imm_]) was calculated from the measured initial rate of oxygen consumption (*r*_O2_) and the concentration of immobilized enzyme (E_imm_) in mg/mL. The same activity assay was applied to the free enzyme when direct comparison of the activities of soluble and immobilized oxidase preparations was needed.

Protein concentrations were determined using the Bradford assay referenced against known concentrations of BSA.

### Determination of stabilities

Stability of free and immobilized preparations of *Tv*DAO at 50°C was assayed in 2 mL round-bottomed microfuge tubes on an Eppendorf Thermomixer with shaking at 500 rpm. A protein concentration of 1 μM in 10 mM Tris-HCl buffer, pH 7.5, or – in the case of the immobilized preparations – a suspension corresponding to this value was used. The pH of the buffer was always controlled and if necessary, adjusted at the temperature of measurement. Buffer was supplemented with 100 μM FAD where indicated. Samples were taken in regular intervals throughout the experiment. To determine the release of FAD cofactor from the immobilized enzyme preparations, 800 μL of the supernatant buffer were withdrawn from a 1 mL sample. The concentration of FAD was then determined using a Hitachi F-4500 fluorescence spectrophotometer (Hitachi, Tokyo, Japan) at 25°C [22]. The remainder of the sample (containing all of the immobilized enzyme particles) was used to measure the residual activity with the oxygen-dependent assay. Activity of the free enzyme was routinely assayed photometrically with DCIP as terminal electron acceptor using a sample volume of 200 μL. Stability curves for the free enzyme obtained using both O_2_- and DCIP-based assays gave consistent results. For FAD measurements, protein was removed from the soluble sample (800 μL) by ultrafiltration using Vivaspin 500 ultrafiltration tubes (Vivascience) and the concentration of free FAD was determined as described above.

Inactivation of the resting enzyme by gas bubbles was performed at 30°C in a miniaturized reactor system described recently [24]. The reactor was equipped with a heat jacket for temperature control and a suspended magnetic stirrer. The stirring rate was set at 300 rpm. Pressurized air was sparged through a teflon tube (internal diameter 1 mm) at a flow rate of 15 L/h into 20 mL of a stirred solution (or suspension) of the respective *Tv*DAO preparation (1 μM protein concentration) in 10 mM Tris-HCl buffer, pH 7.5. Samples of 1 mL were taken from the reactor using a syringe and the activity was assayed as described above. The non-ionic detergent Pluronic F-68 (Sigma, St. Louis, MO) was added to a final concentration of 1% where indicated. Time courses of activity were corrected for eventual evaporation during the experiment. Control experiments at 30°C without aeration were performed like the stability measurements at 50°C.

Operational stability of the different *Tv*DAO preparations was determined using the integral method described by Nahalka et al. [24]. A substrate concentration of 20 mM D-methionine was chosen; the enzyme concentration was 1.0 μM for *Tv*DAO immobilized on Sepabeads, 0.5 μM for the *Strep*-immobilizate and 0.2 μM for the free enzyme. All other conditions in the reactor were as described above. Instead of the fiber-optic oxygen sensors used for the activity measurements, a mechanically more robust micro-oxygen electrode (model MI-730, Microelectrodes, Inc., Bedford, NH) was used to follow the concentration of O_2 _during the reaction. The electrode was connected via an amplifier to an UDAS-1001E Data Acquisition System (Intelligent Instrumentation Inc., Tucson, AZ) interfaced to a laptop computer through the USB-port. Acquisition, display and storage of the oxygen data was done using a self-written program that had been created with Visual Designer Application Generator (Intelligent Instrumentation).

In cases where the biocatalyst was stable for a longer period of time, substrate was added regularly in solid form to ensure that its concentration was saturating throughout. The time-points for addition of substrate were determined in preliminary experiments. Due to their smaller size the *Strep*-Tactin MacroPrep particles were carried to the surface of the liquid by air bubbles where they tended to adhere to the walls of the reactor. The frequent manipulation required to rinse the particles down caused major irregularities in the response of the oxygen sensor which had to be eliminated from the time courses of dissolved O_2_. The *k*_L_*a*-values for the different conditions used were determined with the gassing-out method.

The stability of the covalently immobilized enzyme was also determined in repeated batches of D-Met conversion where 20 mM initial substrate concentration was oxidized under the conditions described above. Each cycle of conversion was initiated by addition of fresh substrate with aeration of the reactor turned off. Thus it was possible to determine the activity of the immobilized *Tv*DAO preparation at the beginning of each cycle from the initial rate of oxygen consumption [24]. Aeration was turned on again, the oxygen concentration was followed and conversion was allowed to proceed until the level of O_2 _had reached a stable equilibrium concentration (approx. 240 μM; Δ[O_2_] ≤ 2 μM/min). Aeration and stirring were stopped, the beads were allowed to settle and the supernatant was then withdrawn from the reactor. The immobilized catalyst was washed and fresh buffer was added to the original volume before initiating a new cycle of reaction. If interruption of the experiment between two batches was necessary, the immobilized catalyst was covered with a small amount of fresh buffer and stored in the reactor at 4°C. The sensor was re-calibrated at the beginning of each new batch; control of the pH value at the end of every conversion showed that the pH had not been altered.

The mechanical integrity of both carriers was controlled using light microscopy after completing the operational stability experiments. Sepabeads EC-EP were fully stable with all particles present in perfect spherical shape; also the *Strep*-Tactin MacroPrep material, which is not originally designed for use in a reactor, exhibited good mechanical stability with only a minor fraction of broken particles.

## Results and discussion

### Immobilization of *Tv*DAO

For immobilization of *Tv*DAO on epoxy-activated Sepabeads EC-EP we followed a protocol suggested by Mateo et al. [25] for the enzyme penicillin G acylase. It consists of three basic steps: hydrophobic adsorption of the enzyme on the beads at high ionic strengths; formation of covalent bonds between enzyme and carrier; and blocking of the free reactive groups of the carrier with an amino acid, which should also render the carrier surface more hydrophilic. After mixing of an enzyme solution with Sepabeads EC-EP in a 10:1 ratio we observed an immediate drop of activity and protein concentration in the supernatant. Activity and protein in solution further decreased to levels that were below the respective detection limit after one hour of incubation. To ensure that adsorption of the enzyme on the carrier reached completion, incubation was continued for three further hours. We then proceeded with immobilization as described in detail under Methods. Note that no unbound protein was detected in any of the buffers or washing solutions.

**Figure 1:**
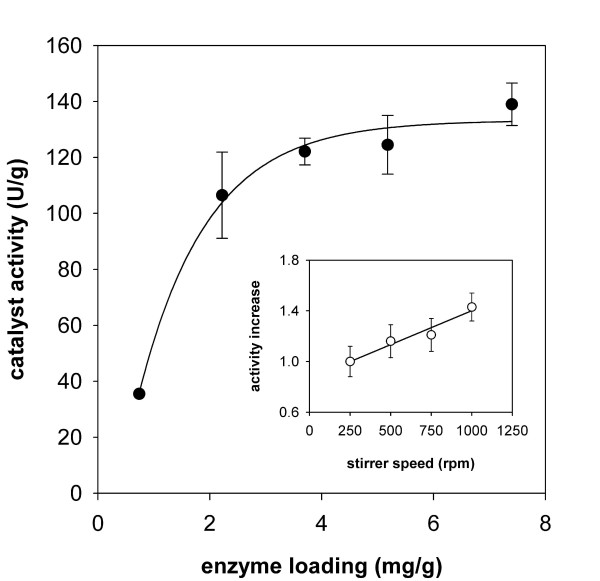
**Effect of enzyme loading on the activity of *Tv*DAO immobilized on Sepabeads EC-EP**. Activity was assayed using measurement of oxygen consumption during oxidative deamination of D-methionine. The solid line shows the trend of the data. Inset: change of the apparent enzyme activity as a function of the stirrer speed used in the O_2_-dependent assay; the solid line is a straight line fit to the data. Error bars show S.D. from three independent assays.

At the enzyme loading routinely used in this work, i.e. approx. 5 mg/g, the specific activity of the immobilized catalyst was 27 ± 3% of the free enzyme. This efficiency could be increased by lowering the enzyme-to-carrier ratio and approached a maximum of 50% at enzyme loadings below 2 mg/g. In this range, the catalyst activity (expressed as U per g carrier) increased almost linearly with the amount of enzyme loaded (Figure 1). The specific activity of the enzyme (expressed as U/mg) thus remained unaltered. However, a clear deviation from this linear relationship was noticed as the amount of enzyme loaded on the carrier was increased to values higher than 2 mg/g (see Figure 1). When protein binding exceeded a value of about 5 mg/g, a further increase in enzyme loading eventually showed only marginal effects on the activity per mass unit of carrier.

The apparent activity of the standard preparation of *Tv*DAO immobilized on Sepabeads (5 mg/g) was also dependent on the speed of stirring in the oxygen-based activity assay. By augmenting the stirrer rate in a stepwise manner from 250 to 1,000 rpm, the apparent activity gradually increased by 40% (Figure 1; inset). These findings clearly indicate that for *Tv*DAO immobilized on Sepabeads the enzymatic reaction is partly limited by diffusion, an effect that is not uncommon for carriers of this type [14].

As a contrast to the covalent immobilization on Sepabeads, which is random in orientation, we have used non-covalent oriented attachment of *Tv*DAO through its affinity tag (*Strep*-Tag II) to *Strep*-Tactin coated on porous MacroPrep particles [23]. Previous work showed that the enzyme bound to the MacroPrep carrier exhibited native-like performance in terms of activation free energy and catalytic efficiency in the oxidative deamination of D-methionine [23], suggesting that diffusion is not limiting the apparent activity of the oxidase. Although oriented immobilization can be done directly from crude cell lysates of *E. coli *expressing *Strep*-tagged *Tv*DAO [23], we preferred using purified enzyme for better control of the protein loading. Note that the amount of enzyme loaded on this carrier was chosen according to the guidelines given by the manufacturer and corresponds to the maximum binding capacity of the *Strep*-Tactin beads.

### Stability of immobilized *Tv*DAO preparations at high temperature

**Figure 2:**
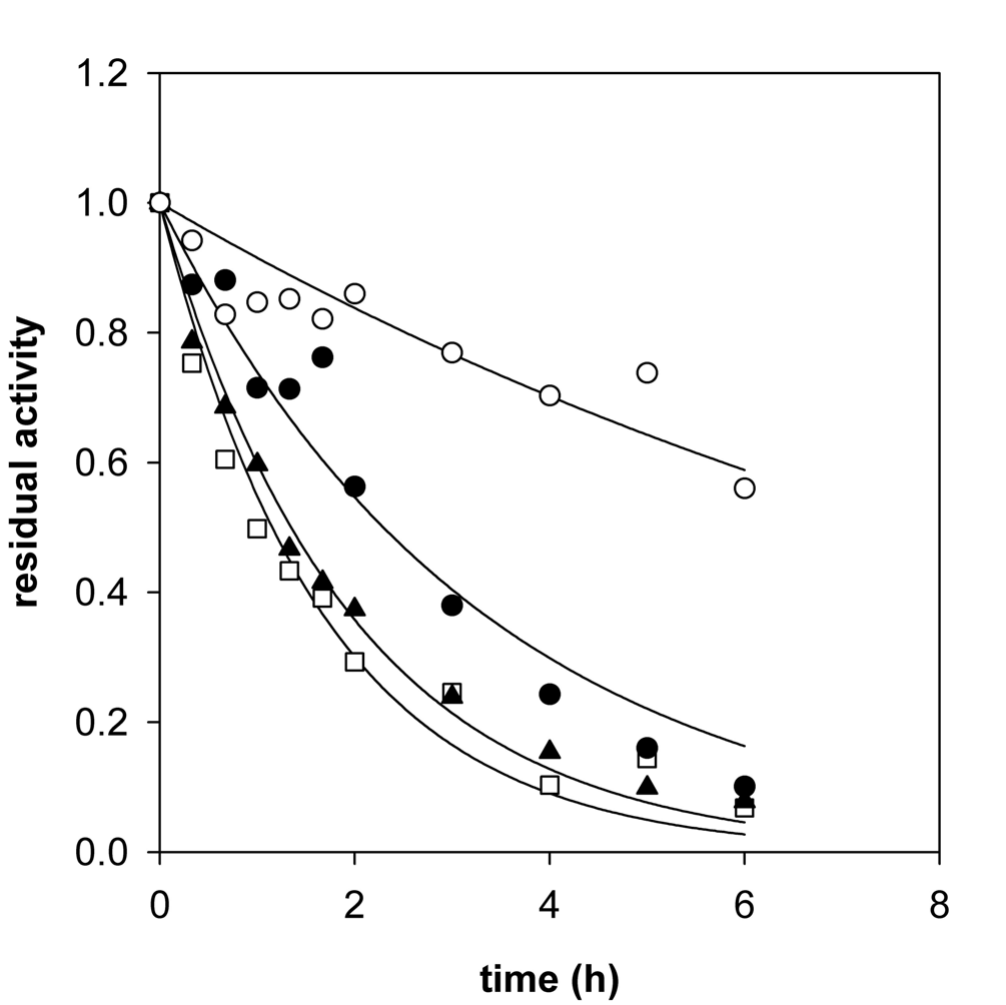
**Thermally induced inactivation of free and immobilized preparations of *Tv*DAO**. Time courses of inactivation of *Tv*DAO immobilized on *Strep*-Tactin MacroPrep material (open squares) and Sepabeads EC-EP (full circles) are compared to the time course of inactivation of the soluble enzyme (full triangles). The inactivation time course of the Sepabeads immobilizate in the presence of 100 μM FAD is shown as open circles. The enzymes were incubated at 50°C on an Eppendorf Thermomixer using orbital shaking at 500 rpm; the protein concentration was 1 μM in 10 mM Tris-HCl buffer, pH 7.5 in all cases. Solid lines represent least squares fits of a single exponential decay function to the data. Note that the suspensions of immobilized biocatalysts were handled like liquids, which explains the observed scattering of the data for the enzyme immobilized on Sepabeads EC-EP.

**Figure 3:**
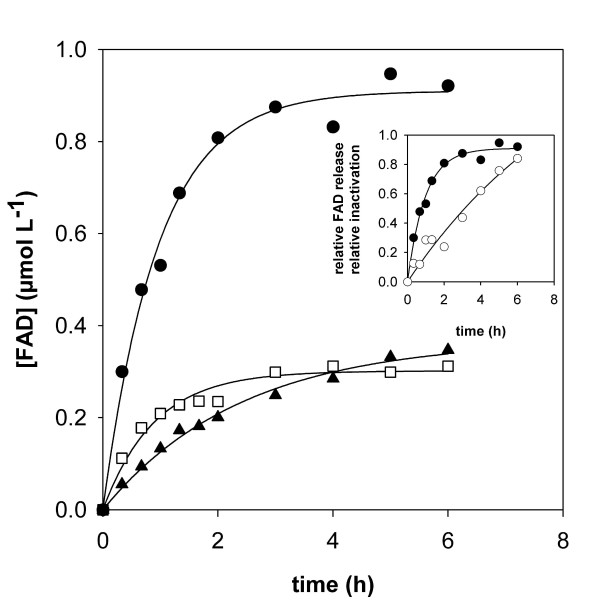
**Release of FAD cofactor during thermally induced inactivation of different preparations of *Tv*DAO**. The concentration of free FAD was measured in samples taken during the course of the inactivation experiment at 50°C. After removal of the enzymes by sedimentation (immobilized preparations) or ultrafiltration (soluble *Tv*DAO) the content of FAD in solution was determined fluorometrically. Symbols are used as in Figure 2; exponential rise functions were fitted to the experimental data (solid lines). The inset compares relative inactivation (open symbols) and relative FAD release (full symbols) for *Tv*DAO immobilized on Sepabeads EC-EP.

Free and immobilized preparations of *Tv*DAO were analyzed with respect to their stability at 50°C. Experiments were performed using an enzyme concentration of 1 μM. The biocatalysts were present under resting conditions due to the absence of an amino acid substrate. Time courses of loss of activity in the different enzyme preparations are compared in Figure 2. Immobilization of *Tv*DAO on the *Strep*-Tactin MacroPrep material did not confer significant extra stability to the oxidase at 50°C as compared to the soluble enzyme incubated under otherwise identical conditions. This result is confirmed by measurement of the release of FAD cofactor from the enzymes (Figure 3) which was – within limits of error – identical in soluble and *Strep*-immobilized *Tv*DAO. Likewise, the stabilizing effect of externally added FAD (100 μM) was virtually identical for the two enzyme preparations (≈1.8-fold for both; data not shown). These findings strongly suggest that the overall mechanism of thermally induced inactivation of *Tv*DAO and the ratio of the two major independent pathways of enzyme denaturation, partial loss of protein structure and release of FAD cofactor [22], were not altered by affinity-based immobilization of the oxidase.

Multi-point covalent attachment of *Tv*DAO on Sepabeads EC-EP caused a significant stabilization of the enzyme activity. The half-life time of the immobilized oxidase (*t*_0.5_; derived from fitting a first-order decay function to the data) at 50°C was 2.3 h and elevated by a factor of 1.7 as compared to the *t*_0.5 _value of the soluble enzyme. However, the stabilizing effect of the immobilization on Sepabeads EC-EP was quite small when compared to what other authors have reported in terms of the stabilization of *Tv*DAO by immobilization (for review, see [15]). Figure 3, in which time courses of dissociation of the FAD cofactor from soluble and immobilized preparations of *Tv*DAO are compared, provides an explanation for this unexpected result. FAD was released into solution by far more rapidly from the immobilized enzyme than from soluble *Tv*DAO. In the case of the carrier-bound oxidase, FAD was completely detached from the enzyme within the timespan of the experiment (Figure 2). However, if on the other hand a 100-fold molar excess of FAD over enzyme was added to the buffer, the immobilized oxidase was stabilized significantly (Figure 2), its half-life now being approximately 8 h. The aggregate data from these experiments indicates that in contrast to the soluble enzyme [22], the release of cofactor has become the predominant pathway of thermally induced denaturation of *Tv*DAO immobilized on Sepabeads. This result receives support from an earlier publication of Betancor et al. [16] who carried out a comparative analysis of the stabilities of immobilized oxidase preparations from *T. variabilis *and *Rhodotorula gracilis*. These authors found that *Tv*DAO was stabilized by external FAD at 42°C and pH 9.0 whereas the inactivation of the enzyme from *R. gracilis *was not affected by addition of the cofactor. However, because of the extremely high concentrations of FAD (50 mM) used in the stability experiments of Betancor et al. [16] it is difficult to distinguish a specific stabilization of *Tv*DAO that results because dissociation of the cofactor from the active site of the enzyme is prevented by the law of mass action when an excess of free FAD is present in solution, from another stabilization where non-specific interactions between the protein and the external FAD are involved. A change in the kinetic mechanism of thermally induced inactivation of *Tv*DAO that is brought about by immobilization of the enzyme on Sepabeads (see Figure 3) is explicable on account of a scenario in which multipoint covalent attachment has, while stabilizing the enzyme through rigidification of its overall structure [15], influenced the binding affinity of the FAD cofactor at 50°C in a negative way.

The inset of Figure 3 compares the time course of FAD release to the corresponding time course of fractional loss of enzyme activity in *Tv*DAO immobilized on Sepabeads. The results reveal that inactivation of the carrier-bound oxidase proceeds markedly slower than one would expect from the rate of FAD dissociation. Therefore, these findings imply that the measured activity of the Sepabeads immobilizate does not reflect the true amount of active enzyme bound to the carrier. They represent yet another good indication that the apparent activity of this insoluble biocatalyst is limited by diffusion.

### Inactivation of *Tv*DAO at the gas-liquid interface

**Figure 4:**
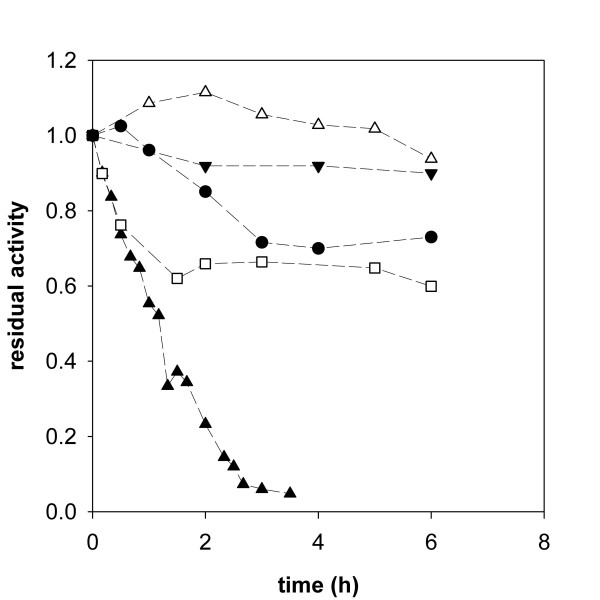
**Interfacial inactivation of free and immobilized *Tv*DAO preparations**. Loss of activity induced by bubble aeration in a miniaturized stirred reactor is shown for the different enzyme preparations and compared to a non-aerated control of soluble *Tv*DAO (full down triangles). Experiments were performed at 30°C with stirring at 300 rpm and an aeration rate of 15 L/h; protein concentration was 1 μM in Tris-buffer. Symbols: full triangles, soluble enzyme; open triangles, soluble enzyme in presence of 1% Pluronic F-68; open squares, *Tv*DAO immobilized on *Strep*-Tactin MacroPrep beads; full circles, *Tv*DAO on Sepabeads EC-EP.

Inactivation at elevated temperature is widely being used to evaluate stability and stabilization of enzymes and is without doubt an appropriate tool to investigate the underlying molecular mechanisms of thermally induced denaturation. Under process conditions, however, the biocatalyst may be exposed to strains different from the stress of elevated temperature. In the case of a reaction that requires the supply of oxygen, efficient aeration is a critical issue in the process. Sparging air into the reaction mixture is fairly simple and efficient and represents the most common method to deliver O_2 _in a reactor. However, DAO from *T. variabilis *has been reported to inactivate at the interface of liquid and gas bubbles [20,27]. Using a miniaturized reactor system with bubble aeration through a sparger tube we could fully confirm and extend these prior findings. Figure 4 shows the inactivation of soluble *Tv*DAO in the aerated reactor in absence of an amino acid substrate. Addition of the non-ionic detergent Pluronic F-68, a surfactant that has been reported to protect enzymes from interfacial inactivation [28], in 1% final concentration, however, led to full stabilization of *Tv*DAO under bubble-aeration conditions.

Figure 4 also shows time courses of inactivation of the two carrier-bound preparations of *Tv*DAO. Although some activity was lost in the immobilized enzymes due to bubble aeration, comparison of data for free and insoluble oxidase preparations clearly emphasizes the positive effect of immobilization on either of the two carriers on *Tv*DAO stability. Interestingly, loss of activity in the immobilized oxidase preparations was observed only in the beginning of the incubation. At later times in the experiment, *Tv*DAO bound on Sepabeads and *Strep*-Tactin MacroPrep material retained a stable level of activity at 70% and 65% of the original value, respectively. Further investigation of the mechanism of this inactivation was beyond the scope of the paper, however, a speculative but plausible interpretation would be that the fraction of enzyme that was bound in the outer spheres of the carrier was exposed to the gas bubbles and thus suffered from inactivation whereas *Tv*DAO bound in the porous core of the material was protected efficiently (for the general case, see [15]).

### Stability under conditions of substrate conversion

**Figure 5:**
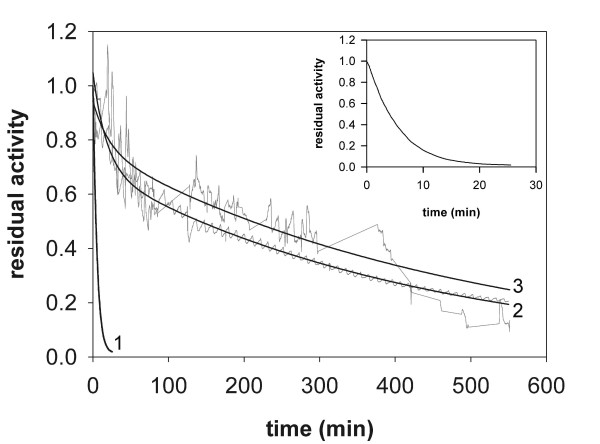
**Stability of *Tv*DAO under conditions of substrate conversion**. The operational stability under bubble aeration conditions is compared for soluble enzyme in absence (1; and inset) and presence of 1% Pluronic F-68 (2) and *Tv*DAO immobilized on *Strep*-Tactin MacroPrep particles (3). The initial concentration of D-methionine was 20 mM and it was added in solid form to the reactor in regular intervals to prevent its depletion. A total volume of 20 mL was used; all other conditions were as described in Figure 4. The black lines for series 2 and 3 show least squares fits of a double exponential decay function to the actual stability curves which are shown in grey.

With bubble aeration identified as a major cause of enzyme inactivation and having at hand efficient ways to protect *Tv*DAO from these deleterious forces, we were interested to see the performance of the different enzyme preparations under operational conditions. Figure 5 (inset) shows that during substrate conversion in a batch reactor, the free enzyme was inactivated even more rapidly than in the absence of an amino acid substrate. It is obvious that apart from the physical stress resulting from bubble aeration, processes immediately associated with the action of the enzyme, e.g. (sub)molecular motion or formation of peroxide [20], led to inactivation here. Addition of Pluronic F-68 (1%) stabilized soluble *Tv*DAO also under conditions of substrate conversion (Figure 5) and may be a highly valuable additive in any reaction employing the free enzyme. However, the fact that the volumetric liquid-phase mass transfer coefficient for O_2 _(*k*_L_*a*) under the conditions used was reduced from 1.46 to 0.91 min^-1 ^due to the addition of 1% of the surfactant provides a note of caution for the use of Pluronic F-68.

In agreement with the good resistance of *Tv*DAO on *Strep*-Tactin MacroPrep beads against interfacial inactivation we observed increased operational stability of this non-covalently immobilized enzyme preparation under bubble aeration conditions. This is certainly remarkable as a similar affinity-based strategy to immobilize DAO from *R. gracilis *has failed to yield a biocatalyst that was stable under operational conditions [29]. Furthermore, the binding of the enzyme to the *Strep*-Tactin support is reversible so that inactivated enzyme could in principle be removed to regenerate the carrier as done in the case of hydrophobic immobilization of *Tv*DAO on Sepharose material [5].

Interestingly, the operational stability of the *Strep*-immobilized enzyme preparation was similar to that of the free enzyme in presence of Pluronic F-68. It also compared well to the operational half-life time of approx. 4 h reported for *Tv*DAO under surface-aerated conditions [24]. Although not unambiguously proven by these results it is tempting to speculate that the stabilization seen here is prevailingly due to efficient protection of the enzyme from interfacial inactivation through its immobilization on a porous support.

**Figure 6:**
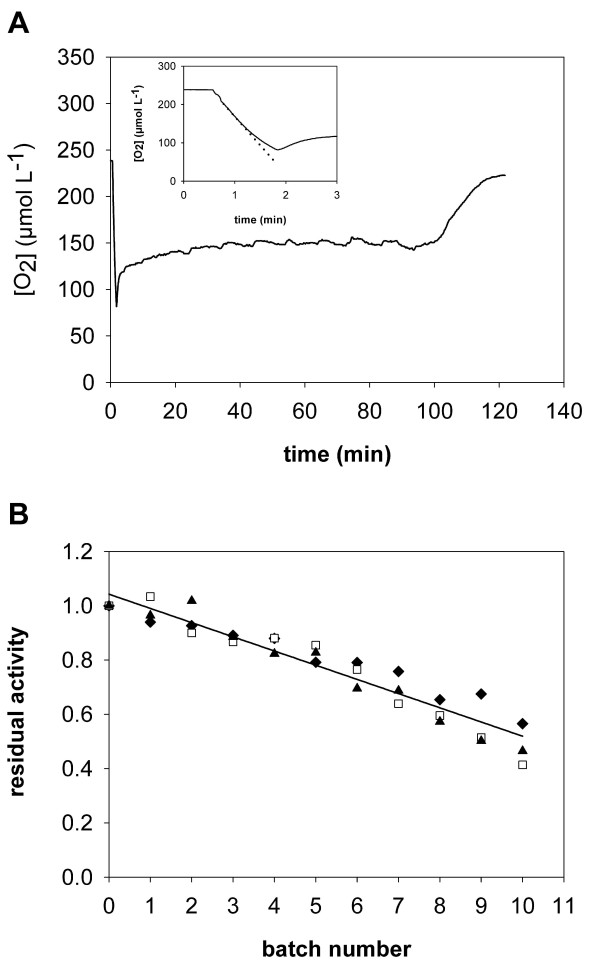
**Stability of *Tv*DAO immobilized on Sepabeads EC-EP in repeated batches of D-Met conversion**. Oxidative deamination of the substrate (20 mM initial concentration in 20 mL Tris-buffer) was done in a stirred reactor (300 rpm) at 30°C with aeration at 15 L/h. Panel A shows a typical time-course of the concentration of dissolved oxygen during one batch of D-Met conversion. The inset illustrates the determination of enzymatic activity at the beginning of each batch from initial rates of oxygen consumption (dotted line) after addition of the substrate. Aeration was turned off for the measurement and turned on again when the level of O_2 _had dropped below 100 μM, which is marked by an increase of the signal. Panel B shows the operational stability of the immobilized enzyme and compares the three methods to calculate the residual activity in every batch: initial rate measurements (triangles); the average oxygen concentration as long as D-Met is not limiting (diamonds); and the reciprocal of the total batch time (open squares). The solid line is a straight-line fit to the averaged data from all three methods.

Under conditions of batch operation with repeated addition of substrate, *Tv*DAO immobilized on Sepabeads EC-EP remained stable over more than 10 h (data not shown). We therefore determined the operational stability of this enzyme preparation in repeated batches of substrate conversion. An initial concentration of 20 mM D-Met was converted under the conditions described above, and oxygen concentration was monitored on-line. Figure 6A shows a typical time course of D-Met consumption. At the beginning of each batch, aeration was turned off before adding the substrate in order to determine the initial rate of substrate conversion (inset of Figure 6A). After turning aeration on again, the level of dissolved O_2 _rose and remained relatively constant as long as the concentration of amino acid substrate was saturating. Substrate limitation was indicated by a sharp increase in oxygen concentration which then approximated the equilibrium value. At this point substrate was considered to be fully depleted, and the reaction was stopped.

Three different parameters can now be used to determine the (residual) activity of the biocatalyst: initial rate measurements at the beginning of each cycle of conversion; the average concentration of oxygen (O_2,av_) in the steady-state phase of the reaction where D-Met was not limiting (note: the value of O_2,av _is determined by the relative rates of oxygen transfer to the bulk solution and enzymatic consumption of dissolved O_2 _[24]); and the reciprocal of the time needed for complete substrate conversion. Figure 6B shows that results obtained with these three different methods of evaluation compare well. It also shows that the immobilized enzyme was highly stable and retained approximately half of its initial activity after 11 cycles of D-Met conversion. Of course, the concept of half-life times in its stern definition does not apply here, but based on the cumulative time after which half of the activity was lost (32.4 h), the stability of *Tv*DAO on Sepabeads was increased by a factor of 480 as compared to the free enzyme (*t*_0.5 _≈ 4 min). Furthermore, this immobilized preparation was markedly more stable than non-covalently immobilized *Tv*DAO or the free enzyme in presence of the surfactant Pluronic F-68. Apparently, immobilization on Sepabeads EC-EP provides an extra stability to the enzyme that goes beyond mere protection from bubble inactivation. Multipoint-covalent attachment is generally known to render the enzyme structure more rigid and might thus reduce molecular motion leading to inactivation of the enzyme during reaction [15].

## Conclusion

We have investigated the stabilization of *Tv*DAO achieved by two different strategies of immobilization, one-point non-covalent immobilization through the affinity of the enzyme's *Strep*-tag to *Strep*-Tactin coated carriers and multi-point covalent immobilization on an epoxy-activated support. At 50°C, the one-point attachment did not stabilize the enzyme against inactivation, a phenomenon also observed for yeast DAOs immobilized on poorly activated glyoxyl agarose [16]. In the case of covalent multi-point immobilization of *Tv*DAO, two antagonistic effects on the stability of the enzyme at elevated temperature seem to be competing. Whereas the overall protein structure was apparently stabilized by multi-point attachment to the support, the binding of FAD cofactor was weakened in the immobilized catalyst. This effect counteracted the stabilizing effect of immobilization such that the overall stability of *Tv*DAO on Sepabeads EC-EP was not markedly different from that of free enzyme. If one could strengthen the cofactor binding in *Tv*DAO through protein engineering, e.g. like this has been achieved for a mutant of human DAO which has the flavin attached covalently [30], and immobilize this enzyme variant by covalent multi-point attachment, a highly thermostable biocatalyst might be generated.

Under process conditions, one of the major factors causing inactivation was identified as denaturation occurring at the interface of liquid and air bubbles. Either one of the immobilization methods used here could stabilize the enzyme against bubble inactivation, probably because the porous structure of the carriers provided protection (for a general discussion, see the review of Mateo et al. [15]). The stabilizing effect of multi-point immobilization, however, goes beyond mere protection of the biocatalyst from gas-liquid interfaces and *Tv*DAO attached on Sepabeads EC-EP clearly outperformed the affinity-based immobilizate. Using a straightforward experimental method that requires the measurement of only one parameter (the concentration of dissolved oxygen) we have determined the operational stability of covalently immobilized *Tv*DAO in repeated batches of substrate conversion. The ease of use and the relatively low requirement for specialized equipment are advantages of the method which could be applied for determining the stability of any oxygen dependent enzyme under conditions of substrate conversion.

## Authors' contributions

I.D. and B.N. designed research; I.D. performed experiments and analyzed data; I.D. and B.N. wrote the paper.
